# Impact of Nutritional Status of Patients with Head and Neck Squamous Cell Carcinoma on the Expression Profile of Ghrelin, Irisin, and Titin

**DOI:** 10.3390/cancers16020437

**Published:** 2024-01-19

**Authors:** Agata Andruszko, Jarosław Szydłowski, Beniamin Oskar Grabarek, Katarzyna Mazur, Tomasz Sirek, Piotr Ossowski, Mieszko Kozikowski, Konrad Kaminiów, Ariadna Zybek-Kocik, Jacek Banaszewski

**Affiliations:** 1 Department of Otolaryngology and Laryngological Oncology, Poznan University of Medical Sciences, 61-701 Poznan, Poland; aandruszko85@gmail.com; 2 Department of Pediatric Otolaryngology, Poznan University of Medical Sciences, 61-701 Poznan, Poland; szydlowski@ump.edu.pl; 3 Department of Medical and Health Sciences, Collegium Medicum, WSB University, 41-300 Dąbrowa Górnicza, Poland; beniamin.grabarek@wsb.edu.pl (B.O.G.); drpiotrossowski@gmail.com (P.O.); konrad.kaminiow@wsb.edu.pl (K.K.); 4 Gyncentrum, Laboratory of Molecular Biology and Virology, 40-851 Katowice, Poland; 5 Faculty of Health Sciences, The Higher School of Strategic Planning in Dąbrowa Górnicza, 41-300 Dabrowa Gornicza, Poland; k.mazur@wsps.pl; 6 Department of Plastic Surgery, Faculty of Medicine, Academia of Silesia, 40-555 Katowice, Poland; drtskierka@gmail.com; 7 Department of Plastic and Reconstructive Surgery, Hospital for Minimally Invasive and Reconstructive Surgery, 43-316 Bielsko-Biała, Poland; 8 Faculty of Medicine, Uczelnia Medyczna im. Marii Skłodowskiej-Curie, 00-136 Warszawa, Poland; mieszko.kozikowski@uczelniamedyczna.pl; 9 Department of Metabolism Endocrinology and Internal Medicine, Poznan University of Medical Sciences, 61-701 Poznan, Poland; azybek@ump.edu.pl

**Keywords:** ghrelin, irisin, titin, methylation, head and neck squamous cell carcinoma

## Abstract

**Simple Summary:**

Cachexia and malnutrition are characteristic of oncology patients, including those with head and neck cancer (HNSCC). Fifty-six patients diagnosed with HNSCC (study group) were included in the study, and seventy patients constituted the control group. We used standardized questionnaires to assess nutritional status and cachexia. In our study, we evaluated whether ghrelin, titin, and irisin can be a useful diagnostic marker, a prognostic marker of cancer development in these patients, and whether the levels of these proteins depend on the nutritional status of the patients. We used molecular biology methods to evaluate the concentration of the selected proteins. The results indicate that the levels of ghrelin, titin, and irisin correlate well with the nutritional and cachexia status of patients with HNSCC. Until now, to our best knowledge, this is the first study assessing the changes in concentrations of these proteins in this type of cancer.

**Abstract:**

The goal of this paper was the evaluation of the changes in the expression profile of irisin, ghrelin, and titin in the carcinoma tissue and in the blood of patients with head and neck squamous cell carcinoma (HNSCC), including determining the profile of their expression in relation to patient nutrition. The study included 56 patients with diagnosed squamous cell carcinoma of HNSCC in the T3 and T4 stages of the disease. Healthy control tissue specimens were collected from an area 10 mm outside the histologically negative margin. In turn, the blood and serum from the control group came from healthy volunteers treated for non-oncologic reasons (n = 70). The molecular analysis allowed us to determine the profile of irisin, ghrelin, and titin methylation, evaluate their expression on the level of mRNA (quantitative Reverse Transcription Polymerase Chain Reaction; qRT-PCR) and protein (Enzyme-Linked Immunosorbent Assay Reaction; ELISA) in the carcinoma tissue and the margin of healthy tissue, as well as in serum of patients in the study and control groups. At the start of our observations, a Body Mass Index (BMI) < 18.5 was noted in 42 of the patients, while six months after the treatment a BMI < 18.5 was noted in 29 patients. We also noted a decrease in the expression of irisin, ghrelin, and titin both on the level of mRNA and protein, as well as a potential regulation of their expression via DNA methylation. There is no convincing evidence that the proteins assayed in the present work are specific with regard to HNSSC.

## 1. Introduction

By the term head and neck cancers, we understand malignant epithelial tumors located in the upper part of the digestive and respiratory tracts, meaning in the oral cavity, throat, larynx, nasal cavity, paranasal sinuses, ears, and salivary glands [1,2]. The group of these cancers depending on their location is characterized by different courses and prognoses [1,2].

Nevertheless, a common trait of theirs is diagnostic and therapeutic difficulties. Despite enormous progress, “head and neck cancers” are most often diagnosed at a stage when the tumor already exceeds the original boundaries of the organ with present metastases to the lymphatic system of the neck (a feature of T-3/4 and/or N+) [3]. Each year, there are over 600,000 new cases of head and neck cancer worldwide, with the dominant locations being the oral cavity and the larynx [4,5]. In Poland, head and neck cancers constitute about 4.5% of all instances of malignant tumors [6]. As far as histology, the dominant cancer is head and neck squamous cell carcinoma (HNSCC) showing a tendency to local infiltration and metastases in the lymphatic system of the neck [5]. HNSCC treatment aims at local elimination while maintaining the best possible functional and cosmetic effects [7]. Significant tumors in the head and neck region often require reconstructive surgery in order to maintain function and aesthetics [8]. Both cancer and extensive surgical procedures trigger the catabolic–anabolic imbalance [9].

Interesting in the context of HNSCC are recent studies suggesting a relationship between blood type and the risk of developing HNSCC. Alexander et al. analyzed the medical records of 61,899 patients diagnosed with HNSCC. The analysis showed that the most common blood type among the analyzed patients was blood type A (45.12%), followed by 0 (24.66%), B (20.26%), and AB (5.16%). Moreover, she stated that a significantly higher risk of throat cancer occurred in people with blood type B; a higher risk of oral cancer occurred in people with blood groups A and AB. In turn, people with blood group O have the highest risk of developing oropharyngeal cancer [10]. Jing et al. assessed the relationship between blood type and the risk of nasopharyngeal carcinoma (NPC) in the Chinese population. These researchers found that people with blood type O have a significantly lower risk of developing NPC, although, at the time of disease onset, these people have lower rates of 3-year overall survival (OS), locoregional recurrence-free survival (LRRFS), and free survival from distant metastases (DMFS). At the same time, no analyzed relationship was found among people with blood group A [11]. In turn, Kakava et al., assessing the relationship between blood type and the risk of HNSCC, showed that the largest percentage of patients diagnosed with HNSCC had blood type A, followed by O, B, and AB, while in the control group the most common blood type was O, followed by A, B, and AB. Interestingly, people with blood group A had a 1.52-fold higher risk of developing HNSCC compared to people with other blood groups [12].

Loss of body mass is a problem for nearly 80% of people suffering from cancers and is considered an inherent element of this disease. Moreover, in oncologic patients, cachexia may occur, meaning a multi-factor syndrome that includes a decrease in food intake and metabolic dysfunctions. There are three stages of cachexia. The first of these is pre-cachexia connected with an unintentional loss of body mass below 5% in a period of the last six months, anorexia, and metabolic changes. The second stage is cachexia, meaning loss of body mass above 5% in a period of the last six months or above 2% in patients with a BMI below 20 or sarcopenia. In turn, the third stage is refractory cachexia, which is resistant to treatment and is connected with an active catabolic process, very low fitness, and an expected survival time of below three months. Patients who are the most susceptible to malnutrition are those with cancers of the digestive system [13,14].

The following forms are used in screening evaluation of patient nutrition: NRS2002, SGA, Nutrition Screening Tool (NST), Mini Nutritional Assessment (MNA), Prognostic Nutritional Index (PNI), Nutritional Risk Index (NRI), or Short Nutritional Assessment Questionnaire (SNAQ) [15,16].

This is possible if there is a threat to oncological devices that will be triggered by energy homeostasis and the factors responsible for its maintenance, which will be available in the future as a target of occurrence. However, in this case the focus is on obese and insulin-resistant individuals [17], and the number of studies focusing on the role and relationship between incoming adipokines and nutritional status in patients with HNSC remains fragmentary [18,19].

Ghrelin, irisin, and titin play distinct roles in the regulation of various phycological processes, and although they are not directly related to each other, each contributes in different ways to the maintenance of homeostasis. While ghrelin, irisin, and titin operate in different physiological contexts, they collectively contribute to the overall homeostasis of the body. Ghrelin helps regulate energy balance and food intake, irisin contributes to metabolic regulation through its role in exercise-induced changes, and titin is crucial for the structural integrity and function of muscles. The intricate interplay of various hormones and proteins, each with its unique functions, is essential for the body’s ability to maintain a stable internal environment [20,21].

Ghrelin (the “hunger hormone”) is a peptide hormone produced by the gastric mucosa of the stomach [22]. First discovered and described in 1999 by Kojima and Kangwa, it is a strong stimulator of growth hormone (GH) secretion from the pituitary [22]. There are two forms of ghrelin, acylated, which is the active form, and deacylated [23]. This endocrine peptide has been labeled as a “survival hormone” as its plasma level rises in response to caloric restriction, psychosocial stress, and cachexia [23]. Ghrelin acts by enhancing food intake and body weight gain [24,25]. Moreover, by enforcing growth hormone (GH), it takes part in growth and repair processes [24,25]. Apart from having a great impact on glucose homeostasis and mediating metabolism, ghrelin is also thought to regulate mood, sleep, learning and memory, gastrointestinal motility, gastric acid secretion, bone metabolism, and cardiovascular function, among many others [24,25].

Irisin, also called the “exercise hormone”, is both an adipokine and a myokine, because it acts in adipose and muscle tissues [26]. Its secretion is induced by physical activity and cold [27,28]. Irisin derives from the cleavage of fibronectin type III domain-containing protein 5 (FNDC5) in skeletal muscles [29]. The more muscle mass and the more exercise, the higher irisin production [30]. The main function of this adipomyokine is converting white adipose tissue into brown adipose tissue, which potentially prevents obesity and metabolic syndrome [31,32]. Therefore, irisin boosts energy consumption, and muscle growth improves insulin sensitivity and glucose tolerance [31,32].

A study among healthy women showed a positive association of irisin concentrations with ghrelin and insulin-like growth factor (IGF-1), among others, which may suggest an effect of growth hormone (GH) on irisin secretion by increasing muscle mass [23]. Ghrelin and IGF-1 appear to be additional predictors of circulating irisin levels. Ghrelin stimulating GH release affects growth and muscle mass [33]. In addition, changing irisin levels with age may be associated with decreasing estradiol levels and thus muscle mass [5]. The positive correlation of irisin levels with glucose, total cholesterol, and GH concentrations, parameters that exhibit impaired energy homeostasis, may confirm the compensatory role of Ir in response to deteriorating lipid and carbohydrate metabolism or the existence of the Ir resistance phenomenon [34].

A giant protein—titin—is a component of the sarcomere in striated muscles. Providing structural integrity to the sarcomere, titin spans between the Z-line and the M-line [35]. Titin contributes to the contraction and also supports the passive stiffness of muscles [36]. This human protein has been used as a biomarker in striated muscle-associated diseases, especially in Duchenne muscular dystrophy [37]. Titin also has a significant potential of becoming a biomarker for exacerbated muscle metabolism in patients with thyroid disorders [38]. Also of note is the fact that titin plays an important role in the influx of T lymphocytes into inflamed tissues, including the tumor microenvironment [39]. It should also be noted that titin is constitutively expressed by human lymphocytes, and therefore, abnormal titin expression appears to be involved in the induction and progression of the tumor process [39].

It is also important to keep in mind that gene expression and consequently protein concentration are dependent on epigenetic modifications. One important modification is the methylation of CpG islands within the gene encoding the protein in question [40,41]. The evaluation of epigenetic modifications in the regulation of gene expression is gaining importance, and a large number of gene methylation patterns are characteristic of the cancers in question. However, in the case of HNSCC, the importance of methylation in the regulation of most genes remains unknown [40,41].

Therefore, taking into account the fact that both HNSCC as well as surgical and supplementary treatment trigger the catabolic–anabolic imbalance [9], as well as the significance of ghrelin, irisin, and titin in the control of metabolism, in further stages of our studies we have decided to evaluate their expression profile on three levels of genetic information flow, i.e., methylation of DNA, mRNA, and protein. Until now, to our best knowledge, it is the first study assessing the changes in concentrations of these proteins in this type of cancer.

Thus, the goal of the present text was the evaluation of the changes in the profile of expression of irisin, ghrelin, and titin and also the evaluation of the methylation profile in the carcinoma tissue as well as the blood of patients with HNSCC depending on their nutritional status before treatment, during treatment, and 6 months after the completion of chemotherapy.

## 2. Materials and Methods

### 2.1. Ethics

This prospective, single-center study was conducted following the guidelines of the Helsinki Declaration and approved by the Bioethics Committee of the Medical University of Karol Marcinkowski in Poznan (644/16). Written informed consent was obtained from all subjects involved in the study.

### 2.2. Subjects

A total of 56 patients (24 women and 32 men) with diagnosed squamous cell carcinoma of HNSCC at stage T3 (36 cases, including 11 women and 25 men) and T4 (20 cases, including 13 women and 7 men) from the Department of Otolaryngology-Head and Neck Surgery Poznan University of Medical Sciences in Poland were qualified for the study. Patients were qualified for surgery based on their medical history and symptoms, as well as medical imaging tests—computed tomography and magnetic resonance. The removed tumor tissue underwent a histopathological examination in order to confirm the initial diagnosis. Healthy control tissue specimens were collected from an area 10 mm outside of the histologically negative margin.

In all patients, radical surgical treatment was used along with reconstructive surgery using free flap transfer and supplementary chemoradiotherapy (CTRT) with the use of ONCOR (Siemens, Munich, Germany) linear accelerator (full dose GG-70 Gy; daily dose 2 Gy using IMRT technique). The average time between surgical treatment and the beginning of supplementary treatment equaled 20 ± 5 days. The 7th edition of the TNM scale was used to assess the disease stage. Clinical characteristics and nutrition status of patients from the study group was presented in the Table 1. We present the graphical abstract to better understand our study design, obtained results, and conclusion.

In turn, in Table 2 nutrition status as well as lifestyle factors, such as alcohol consumption, smoking, physical activity, and comorbidities/disease in the study group are presented. Alcoholic status was categorized as social or never drinkers versus former or current alcoholics, defined as having greater than 2 drinks per day. The statistical analysis performed (chi-square test) showed no association between patients’ nutritional status, lifestyle habits, and disease progression (Table 2; *p* > 0.05). The presence of comorbidities was determined by analyzing the patients’ medical records and medical histories.

#### Nutritional Status of Patients Qualified for the Study Group

Apart from the fact that all patients from the study group were evaluated using the Nutritional Risk Score (NRS) scale based on NRS-2002, account age, malnutrition, and disease severity were also taken into account. Based on the evaluation, the patient could have received between 0 and 7 points, out of which 0 to 3 were given in the “nutrition scores” section and an additional 0 to 3 in the “disease severity score”. An additional point was awarded to patients over the age of 70. Those who received at least 3 points were qualified as patients at high nutritional risk upon hospital admission [42].

All patients were also evaluated for nutrition based on the Subjective Global Assessment (SGA) scale, which allowed us to obtain information regarding body mass, food intake, gastrointestinal symptoms, and changes in functional capacity as well as physical examination. A three-point evaluation system was used, where 0 points equaled no dysfunctions, 1 point mild dysfunctions, 2 points moderate dysfunctions, and 3 points severe nutritional dysfunctions. This allowed us to separate the patients into three groups: well-nourished (A), moderately undernourished—pre-cachectic (B), and severely undernourished—cachectic (C), according to the SGA scale. The SGA and NRS evaluations were conducted at three separate time intervals: upon admission to the hospital, before the surgical procedure (T0); prior to CTRT (T3); and 6 months after the completion of CTRT (T5). During T1, T2, and T4, only molecular analyses were carried out.

### 2.3. Control Group

On the other hand, blood and serum from the control group came from healthy volunteers (42 men and 38 women) treated in the Otolaryngological Clinic for non-oncologic reasons (n = 70).

### 2.4. Tissue Collection

Tissues altered by cancer and collected from the margin of changes were secured for further molecular analyses in RNAlater solution (Thermo Fisher Scientific, Waltham, MA, USA; catalog number AM7020) and stored at −80 °C until the start of molecular analyses.

### 2.5. Blood Samples

Whole blood was collected from patients into PAXgene Blood RNA Tubes to secure the material for analysis on the level of mRNA or into PAXgene Blood DNA Tubes in order to secure the material for DNA analysis (methylation). In addition, blood samples were stored in clot tubes, and then they were centrifuged in order to obtain serum to evaluate the concentration of selected factors on the level of proteins.

Blood for molecular analysis was collected before the surgical procedure (T0), on the 1st (T1) and 7th days after the procedure (T2) as well as before (T3), immediately after the completion of CTRT (T4), and finally 6 months after CTRT (T5).

### 2.6. RNA Isolation

The total ribonucleic acid (RNA) extraction from the tissues was carried out using the TRIzol reagent (INvitrogen Life Technologies, Carlsbad, CA, USA, catalog number 15596026) following the manufacturer’s protocol.

In turn, RNA extraction from blood was made using the commercially available PAXgene Blood RNA Kit (Qiagen, Venlo, The Netherlands, Cat No./ID: 762174) according to the manufacturer’s recommendations.

The RNA extracts were evaluated via the analysis of 18S ribosomal RNA (rRNA) and 28S rRNA (agarose electrophoresis) and by analyzing RNA concentration (260 nm) and purification (absorbance ratio 260 nm/280 nm).

### 2.7. Quantitative Reverse Transcription Polymerase Chain Reaction (qRT-PCR) Analysis

The SensiFast SYBR No-ROX One-Step Kit (Bioline, London, UK) was used to assess the expression pattern of *FINDC5* (*irisin*), *ghrelin* (*GHRL*), and *TNT* (*titin*) using a DNA Engine Opticon detector system (MJ Research Inc., Watertown, MA, USA). The thermal profile included reverse transcription (45 °C, 10 min), polymerase activation (95 °C, 2 min) and 40 cycles of denaturation (95 °C, 5 s), hybridization (60 °C, 10 s), and elongation (72 °C, 5 s). Β-actin was used as an endogenous control of the reaction. Primer sequences are listed in Table 3. Results were shown by using the 2^−∆∆Ct^ method (fold change = 1—control; >1—overexpression; <1—silencing). Three technical repetitions were performed for each biological replicate.

### 2.8. Methylation Profile of Irisin, Ghrelin, and Titin

In the first stage, we determined the location of the CpG islands in the sequence of the analyzed genes, i.e., irisin (NCBI Reference Sequence: NM_001171941.3), ghrelin (NCBI Reference Sequence: XM_046926185), and titin (NCBI Reference Sequence: X69490.1). Then, with the use of the MethPrimer program (http://www.urogene.org/cgi-bin/methprimer/methprimer.cgi; accessed 29 January 2022), starters were designed allowing for the detection of methylated and non-methylated sequences using the PCR method with the following assumptions: CpG island length > 100 nucleotides, >50% GC content, and the relation between the observed and expected value > 0.6.

In order to assess the methylation of the evaluated genes after performing the conversion reaction with the use of sodium sulfate VI (bisulfite conversion) in accordance with the recommendations and the purification of the extract, a methylation-specific PCR (MSP) was conducted using the QuantiTect SYBR Green PCR Kit (Qiagen GmbH, Hilden, Germany), and the primers are shown in Table 4. The thermal profile of the reaction was as follows: 95 °C initial denaturation for 5 min, 40 three-step cycles (30 s each); 94 °C denaturation, 65 °C primer annealization; and 72 °C elongation. After the amplification of irisin, ghrelin, and titin, the obtained products were separated using electrophoresis on 1% of agarose gel with added ethidium bromide (final concentration 0.5 µg/mL) in a buffer 1× TBE with a voltage of 120 V. The analysis of the separated fragments was carried out with the presence of pBR322/HaeIII as the size marker. The accuracy of the amplification was confirmed using positive control (DNA methylated) and negative control (non-methylated DNA) using the EpiTect Control DNA (Qiagen GmbH, Hilden, Germany) set.

### 2.9. Enzyme-Linked Immunosorbent Assay Reaction

Changes in the concentration of irisin, ghrelin, and titin in cancerously altered tissues, healthy tissue, and the serum of patients were determined with the ELISA test using the following kits: Human Irisin (IS) ELISA Kit (MyBioSource, Inc., San Diego, CA, USA, catalog number MBS108987), Human Acylated ghrelin ELISA Kit (MyBioSource, Inc., San Diego, CA, USA, catalog number MBS283141), and Human titin ELISA Kit (MyBioSource, Inc., San Diego, CA, USA, catalog number MBS913885) following the manufacturer’s recommendations. Three technical repetitions were performed for each biological replicate. An Infinite M200 PRO microplate reader (Tecan, Männedorf, Switzerland) was used to evaluate absorbance at 540 nm.

### 2.10. Human Papillomavirus Genotyping

Detection of HPV from blood samples was performed using real-time polymerase chain reaction (RT-PCR) based on a single-plate test for genotypes 16 and 18, as recommended by Lindh et al. [43] and Igerslev et al. [44]. RT-PCR was performed on a Roche^®^LC480 Lightcycler.

### 2.11. Statistical Analysis

Statistical analysis was conducted using the Statistics 13PL software (Statfoft, Cracow, Poland) assuming a statistical significance threshold (*p*) below 0.05. To evaluate the normalcy of data dispersion, we used the Shapiro–Wilk test, and based on the obtained results the remaining analysis was conducted using parametric methods—*t*-Student test and one-way ANOVA variance with post-hoc Tukey’s test. The qualitative data were analyzed using the chi-square independence test.

## 3. Results

### 3.1. Nutritional Status of Patients with HNSCC before Surgery, on the Day of the Start, and after 6 Months of Chemotherapy

In the first stage, on the basis of a questionnaire survey, we analyzed the nutritional status of the patients at three time intervals, that is, before surgery, at the time of eligibility for the study (T0); at the time of the start of chemotherapy (T3); and 6 months after the end of chemotherapy (Table 5). The statistical analysis performed did not show the occurrence of a relationship between the nutritional status of the patients and the period of observation carried out (Table 5; *p* > 0.05). Regardless of the period of observation, the largest percentage of patients was moderately malnourished—pre-cachectic (B). In contrast, the percentage of severely malnourished—cachectic patients—was initially 20% and gradually decreased to 14% at 6 months after the end of chemotherapy. We also noted an increase in the percentage of patients who were well nourished (A), from 14% at the beginning of follow-up to 27% 6 months after the end of treatment (Table 5).

### 3.2. The Expression Profile of Genes Coding Irisin, Human Acylated Ghrelin, and Titin in Tumor Extracts and on the Margin of Healthy Tissues and Blood Samples Determined Using qRT-PCR

Based on the conducted statistical analysis we have shown, there was a statistically significant decrease in the expression of irisin, ghrelin, and titin in the cancerous tissue in comparison with healthy tissue from the tumor margin (*t*-Student test; *p* < 0.05).

**Figure 1:**
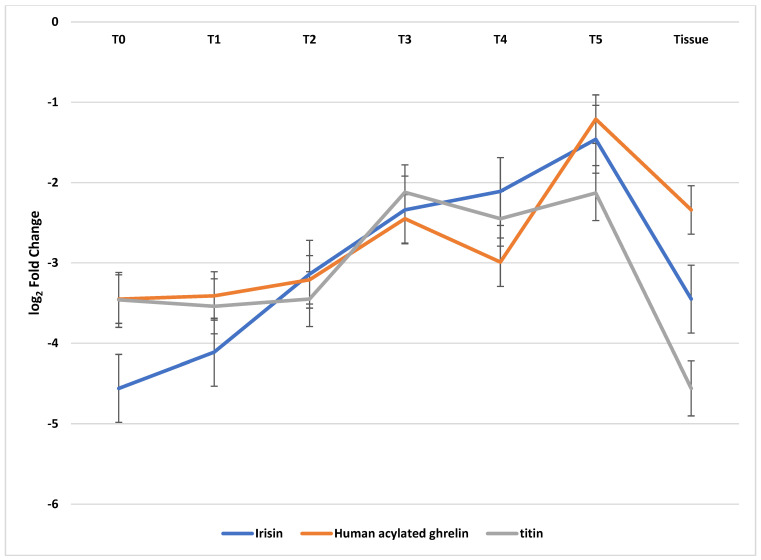
Variances in the expression patterns in the irisin, ghrelin, and titin at the mRNA level in patients with HNSCC in blood and cancer samples.

Apart from that, we showed that changes in the expression patterns of all three of the evaluated mRNAs in the patients’ blood show that they undergo a statistically significant change in comparison with the control (ANOVA one-way variance; *p* < 0.05). A statistically significant silencing of the expression of irisin, ghrelin, and titin in the study group shown with the post-hoc Tukey’s test takes place between T0 and T3 (*p* < 0.05), T0 vs. T4 (*p* < 0.05), and T0 vs. T5 (*p* < 0.05). Moreover, we confirmed the incidence of statistically significant dependencies between the expressions of irisin, ghrelin, and titin at a given point in time, between all three groups (A–C), which differ in their nutritional conditions; the higher the malnutrition level is, the lower the expression of the aforementioned genes (*p* < 0.05). Detailed results of the qRT-PCR are presented in Table 6 and in Figure 1.

### 3.3. Irisin, Ghrelin, and Titin Methylation Profile

Evaluation of the profile of methylation for the evaluated genes shows that in the case of cancerous tissue, there is an epigenetic silencing of the assessed genes by methylation. Similar results were obtained in the case of whole blood samples. In this case, and also in the case of cancerous samples, there is a silencing in the expression of irisin, ghrelin, and titin, in comparison with the control. Since, in all the cancerous samples, we showed methylation for every gene, we did not divide the results based on patient nutrition (Table 7).

### 3.4. The Concentration of Irisin, Ghrelin, and Titin Was Determined Using the ELISA Test in the Tissues and Serum of Patients with HNSCC in Comparison with the Control

In the last stage, we evaluated changes in the profile of concentrations of irisin, ghrelin, and titin proteins in the biopsy specimens collected during the surgical procedure in comparison with the healthy tissue, as well as in the serum of patients during observation. Similar to the mRNA level, we found a significantly lower concentration of the analyzed proteins in the cancerous tissue in comparison with the control tissue (*t*-Student test; *p* < 0.05). We also demonstrated a statistically significant decrease in the expression of these three proteins in the cancerous tissue with various levels of nutrition (A–C; Table 7; *p* < 0.05).

Similarly, also in the serum of patients from the study group, the concentration of irisin, ghrelin, and titin was statistically significantly lower than in the control group independent of observation time (*p* < 0.05). There was also a statistically significant silencing of the expression of irisin, ghrelin, and titin in the study group shown using the post-hoc Tukey’s test between T0 and T3 (*p* < 0.05), T0 vs. T4 (*p* < 0.05), and T0 vs. T5 (*p* < 0.05). In addition, statistical analysis showed statistically significant changes in the expression of irisin, ghrelin, and titin in the serum of oncologic patients depending on the level of nutrition (*p* < 0.05). Detailed results of the changes in the concentrations of evaluated proteins are shown in Table 8.

### 3.5. Assessing the Risk of HNSCC According to Blood Type

In the last stage of our study, we analyzed the frequency of occurrence of individual blood groups among patients and control group participants along with an assessment of the risk of developing HNSCC (Table 8). The analysis showed that the most common blood group in the study group was group A (61%), followed by B (11%), 0 (23%), and AB (5%). In turn, in the control group, blood type 0 predominated (49%), followed by A (38%), B (%), and AB (4%). The statistical analysis performed showed that people with blood type A have a 2.46 times higher risk of occurrence of HNSCC, with group B and AB 1.28 and 1.26, respectively, having a higher risk, and blood group 0 reduces the risk of HNSCC (Table 9).

## 4. Discussion

According to the World Health Organization (WHO), malnutrition is defined as “an imbalance between the need and intake of essential nutrients, which allows for an increase and maintenance of life functions and the performance of specific functions” [45].

It must also be remembered that the connection between inappropriate nutrition and cancer has a bidirectional character. On one hand, the incidence of cancers leads to changes in the nutritional condition, and on the other, its dysfunction negatively influences the patient’s treatment and recovery [46].

In our study, we observed that patients with HNSCC are undernourished, and most of them were included in a group with pre-cachexia or cachexia. Only eight of them (14%) exhibited the appropriate nutritional condition at the moment of inclusion into the study. Although we did note a change in the number of patients assigned to the three groups based on nourishment during the course of our observations, it does not seem that after 6 months of observations, fifteen well-nourished patients (27%) is a satisfactory result.

Nevertheless, it must be taken into account that the evaluation of organism nutrition is a multi-factor parameter, which in order to be understood must involve the combination of various methods and tools. That is why those that are most often used are the simplest ones, the ones that allow for rapid results, meaning nutritional assessment/risk of undernourishment questionnaires and anthropometric measurements. On the other hand, the more complicated methods include biochemical and molecular tests, as well as additional examinations [47,48,49,50].

The cancerous process is inherently connected with a chronic inflammatory condition during which there are changes in the metabolism of carbohydrates, lipids, and proteins [51]. Loss of body mass in undernourished and cachectic patients is connected with an increase of lipolysis in white adipose tissue and anorexia [51].

Both in the cancerous tissue and the serum of patients with HNSCC we noted a significantly lower concentration of these three adipokines. Apart from that, in patients with more clinically advanced HNSCC (T4), their concentration was lower than in patients with HNSCC of lesser severity (T3).

Provatopoulou et al. noted a significantly lower concentration of irisin in the serum of women with breast cancer in comparison with the control, while both in the study group as well as in the control group the BMI level was correct. These authors did not observe BMI < 18.5 [52].

Taking into account the observations made by Gannon et al. based on an in vitro model of breast cancer, when irisin is added to the cell culture apoptosis of cancer cells increases while the migratory potential of these cells decreases and the nuclear factor kappa beta (NFκB) signaling pathway is suppressed [53]. Similar findings were made by Shao et al., who also showed that irisin inhibited Epithelial–Mesenchymal Transition (EMT) via the PI3K/AKT/Snail pathway [54]. In turn, Moon et al., using both human and mouse in vitro models of obesity-related cancers such as endometrial (KLE and RL95-2), colon (HT29 and MCA38), thyroid (SW579 and BHP7), and esophageal (OE13 and OE33) cancers, did not show that irisin in a range of concentrations between 5 nmol/L to 100 nmol/L had any effect on the migration of cancer cells [55]. Nevertheless, the conclusions from the study of Moon et al. are not confirmed by any other study and are a result of a different methodology of the conducted studies [55]. When Spiegelman et al. discovered irisin and its biological properties [56], an attempt was made to determine the physiological range of its concentrations during the resting state and after physical activity. Jędrychowski et al. showed an increase in the concentration of irisin in physically active persons in comparison with people who led a sedentary lifestyle (4.3 ng/mL vs. 3.6 ng/mL; *p* < 0.05) [57]. Moreno et al. also showed that the concentration of irisin is higher for people who are physically active than in those who lead a sedentary lifestyle (128.55 μg/mL ± 78.71 μg/mL vs. 105.66 μg/mL ± 60.2 μg/mL; *p* < 0.05) [58]. The differences in the concentrations of irisin noted by Jędrychowski et al. and Moreno et al. may have resulted from the fact that different methods of detection were used to assess the concentration of irisin. Jędrychowski et al. used mass spectrometry [57], while Moreno et al. utilized the ELISA test [58], in which there is a possibility of non-specific binding of antibodies directed against irisin with plasma proteins. Furthermore, as can be seen from observations made by Kim et al., made on a mouse model, the half-life of irisin is shorter than one hour [59]. If the half-life of irisin in the human organism is similar to that of mice, this can have a significant influence on the concentration of irisin in the serum [59]. Our studies indicate that the decreased concentration of irisin in the cancerous tissue and blood serum is connected not only with the cancerous process itself but also with the loss of body mass, including, of course, muscle tissue of patients included in the study. Such a conclusion seems correct since we have observed a statistically significantly lower expression of mRNA and concentration of irisin protein along with progressing cachexia (*p* < 0.05). However, Homa-Mlak et al. also evaluated changes in irisin concentration in patients with head and neck cancers (HNCs) and connected it with the nutrition levels of patients. They noted a higher concentration of irisin in patients with a high risk of undernourishment (NRS > 3) in comparison to patients with a low or moderate risk as well as those who were well nourished [60]. These results are different from ours for several reasons. Firstly, we use a different set of reagents to mark the concentration of irisin in the patients’ serum. Secondly, only patients with advanced cancer on the level of T3 and T4 took part in the study, and only one sort of therapy was used, different than in the study by Homa-Mlak et al. Apart from that, in the study by Homa-Mlak et al. a BMI < 18.5 was observed for only five patients (10%) [60], which is opposite of our study. Similar results to those obtained by Homa-Mlak et al. [60], and therefore different from ours, were noted by Panagiotou et al. [61], Shahidi et al. [62], and Altay et al. [63]. On the other hand, Quiñones et al., in an animal model, did not confirm the relationship between concentration of irisin and nutrition. Nevertheless, to fully understand changes in the concentration of irisin in various pathological conditions it would be necessary to follow the transduction signal induced by irisin [64].

In our study, we have also shown the silencing in the expression of ghrelin, which is another protein we assessed, both on the level of mRNA and the protein, in the clinical material obtained from the study group in comparison to the control, both in the cancerous tissue as well as in the serum. Despite numerous texts regarding the role of ghrelin in the metabolism of glucose; induction of obesity; and skeletal, inflammatory, and aging metabolism, its role in carcinogenesis is inconclusive [65]. It seems that the difficulty in determining and attributing a clear role to ghrelin in physiological and pathological conditions may result in the fact that as a result of collecting a sample for testing, processing it following the recommendations of the manufacturer, and the analytical technique used, a proteolytic transformation of acetylated ghrelin into non-acetylated ghrelin takes place. Blatnik et al. assume that the non-acetylated ghrelin marked in the plasma is a result of the conversion to the acetylated form after sample collection [66]. It must be remembered that only acetylated ghrelin is biologically active [67]. Pritchett et al. assessed the concentration of ghrelin in the serum of patients from the Chinese population with diagnosed esophageal squamous cell carcinoma (ESCC), gastric cardia adenocarcinoma (GCA), and gastric non-cardia adenocarcinoma (GNCA) in comparison with a control group. These authors observed that a lower concentration of ghrelin was connected with a significantly higher risk of development of GNCA and GCA. At the same time, a lower risk of development of ESCC was connected with a lower ghrelin concentration [68]. Another important aspect in the context of our studies is the fact that in studies conducted with populations of Western countries, a positive correlation between low ghrelin concentration and the incidence of gastric adenocarcinoma, esophagogastric junctional adenocarcinoma, esophageal squamous cell carcinoma, and colorectal adenocarcinoma was confirmed [69,70]. A lower ghrelin concentration in the serum of patients from the study group in comparison with the control may be a result of the fact that a significant portion of patients with HNSCC declared that they are both active alcohol drinkers and cigarette smokers, which are factors irritating the mucous membrane of the stomach and that have a potential connection with the development of chronic gastritis, *Helicobacter pylori* infection, and thus to a certain degree an induction of cancerous changes in this organ [71,72]. This is even more relevant since nearly 79% of the ghrelin released into systemic circulation is produced in the stomach [73]. Therefore, it seems that a lowered expression of ghrelin in our patient population may be for the most part connected with the atrophy of stomach cells able to synthesize it. Another factor influencing the results was the fact that a significant number of the patients were severely undernourished. This is confirmed by observations of Zeng et al., who, in a mouse model, showed that the administration of both acetylated and non-acetylated ghrelin to mice resulted in the suppression of the degradation of myofilaments [74]. Therefore, it may be assumed that in oncologic patients ghrelin supplementation could alleviate the symptoms of cancer cachexia [74]. Moreover, Batury et al. showed that methylation of the DNA promoter region of ghrelin is a characteristic of patients devastated by anorexia [75]. Wolf et al. also showed an increase in the level of ghrelin methylation in patients 6 months after bariatric surgery in comparison with the period prior to the operation [76], which confirms our assumptions and results.

The last of the assessed proteins was titin, for which we have also noted a decrease in the study group in comparison with the control. Despite the fact that changes in the expression profile of titin were analyzed above all in the context of muscular metabolism, including Duchenne muscular dystrophy [37], the connection of this protein with carcinogenesis has been studied recently [77]. Su et al. point to the diagnostic utility of evaluating polymorphic variations and mutations in the gene coding titin and using this knowledge in the context of immunotherapy of patients suffering from non-small cell lung cancer [78]. In turn, Han et al. have recently shown that the assessment of the mutation in the *TTN* gene may be used as an unfavorable prognostic marker in thyroid cancer [79]. However, as of yet, we have not found studies assessing changes in the titin profile in oncologic patients in comparison to the control. That is why it is difficult to determine whether titin may become a useful diagnostic or prognostic marker in HNSSC. However, it seems that the decrease in the expression of titin, which we noted in the study group in comparison to the control, is connected with patient cachexia and malnutrition. Therefore, further studies are necessary.

In addition, our observations are also consistent with the results of other studies that find that people with blood type A have the highest risk of HNSCC, while those with blood type 0 have the lowest. The disproportion in the percentage of subjects classified in the study and control groups versus blood type is also apparent. Thus, it seems that blood type also determines susceptibility to HNSCC, and thus people with blood type A should take special care regarding cancer prevention, including in the context of HNSCC. The present observations provide an interesting starting point with regard to the search for complementary predictive markers in oncology [10,11,12].

Our work has both strengths and weaknesses. Its strengths include a high number of patients with HNSCC who qualified for the same treatment, as well as the time of the conducted observations. Secondly, in our study, we have not only taken into account the clinical and anthropometric assessment of patients but have also carried out the molecular analysis of three proteins, which until now have not been studied in the context of HNSCC. On the other hand, the first limitation of our study is its single-facility character. Most likely, taking into account a greater number of facilities would allow us to increase the number of patients in the study group. Secondly, it would be valuable to assess changes in expressions of the evaluated proteins depending on the HNSCC therapy used. Thirdly, it would be beneficial to later conduct a wider analysis of the metabolic conditions of HNSCC patients, including marking other adipocytokines.

Nevertheless, our study is beneficial and is a valuable starting point for further studies.

## 5. Conclusions

The decrease in the expression of ghrelin, irisin, and titin on the level of mRNA and proteins, which we have noted, as well as determination of the status of methylation of those genes in combination with available data from the literature show that the observed expression profile of ghrelin, irisin, and titin is above all a result of cancer cachexia and malnutrition. There is no convincing evidence that the proteins assayed in the present work are specific to HNSSC. However, it seems that changes in the expression profile of ghrelin, irisin, and titin correspond to the nutritional status of patients and may constitute an additional molecular marker in predicting the effectiveness of adjuvant treatment aimed at counteracting body wasting and malnutrition in patients with HNSCC.

## Figures and Tables

**Table 1:** Clinical characteristics and nutrition status of patients from the study group.

Factor	Study Group (n = 56)
Gender	
Male	24 (43%)
Female	32 (57%)
T stage	
T3	36 (64%)
T4	20 (36%)
N stage	
Nx	2 (3%)
N0	5 (9%)
N1	7 (12%)
N2	38 (68%)
N3	4 (8%)
M stage	
Mx	1 (2%)
M0	49 (88%)
M1	6 (10%)
Performance status (PS)	
Disease stage	
I	0
II	2 (4%)
III	15 (27%)
IVA	31 (55%)
IVB	5 (9%)
IVC	3 (5%)
0	0
1	41 (73%)
2	14 (25%)
3	1 (2%)
4	0
Human Papilloma Virus (HPV)-16 status	
Positive	43 (77%)
Negative	13 (13%)
Human Papilloma Virus status-18	
Positive	41 (73%)
Negative	15 (17%)

T, tumor site and size; N, regional lymph node involvement; M, presence or otherwise of distant metastatic spread; HPV, Human Papilloma Virus; Group A, well-nourished, group B, moderately undernourished, group C, severely undernourished.

**Table 2:** Characteristics of the lifestyle factors and comorbidities/disease in the patient study group.

Factor	Study Group (General)	T3	T4	*p*-Value (Chi-Square Test)
Alcohol consumption	25 (69%)	18 (50%)	7 (35%)	0.28
Social	20 (80%)	15 (42%)	5 (25%)	0.64
Former alcoholic	3 (12%)	2 (6%)	1 (5%)	
Current alcoholic	2 (8%)	1 (3%)	1 (5%)	
Never	31 (55%)	18 (50%)	13 (65%)	
Smoking status				
Non-smoker	14 (25%)	12 (33%)	2 (10%)	0.16
Current smoker	35 (63%)	20 (56%)	15 (75%)	
Former smoker	7 (12%)	4 (11%)	3 (15%)	
Nutritional status (parenteral nutrition)				
Yes	15 (27%)	11 (31%)	4 (20%)	0.39
No	41 (73%)	25 (69%)	16 (80%)	
Weight (kg; mean ± SD)	54.65 ± 8.98	54.11 ± 9.18	55.19 ± 10.19	
Body Mass Index (BMI) mean ± SD	20.93 ± 3.12	20.76 ± 3.98	21.09 ± 4.56	
<18.5	38 (68%)	25 (69%)	13 (65%)	0.73
18.5–24.99	18 (32%)	11 (31%)	7 (35%)	
>25	0 (0%)	0 (0%)	0 (0%)	
SGA				
A	23 (41%)	16 (44%)	7 (35%)	0.79
B	20 (36%)	12 (33%)	8 (40%)	
C	13 (23%)	8 (23%)	5 (25%)	
NRS				
1	8 (14%)	4 (11%)	4 (20%)	0.38
2	9 (16%)	7 (18%)	2 (10%)	
3	32 (57%)	22 (59%)	10 (50%)	
>3	7 (13%)	3 (12%)	4 (20%)	
Comorbidities/disease	27 (48%)	17 (47%)	10 (50%)	0.84
Diabetic mellitus (yes)	5 (9%)	3 (8%)	2 (10%)	0.83
Hypertension (yes)	3 (5%)	2 (6%)	1 (5%)	0.93
Hypercholesterolemia (yes)	1 (2%)	0 (0%)	1 (5%)	-
Diabetic mellitus plus hypertension	10 (18%)	7 (19%)	3 (15%)	0.68
Diabetic mellitus plus hypercholesterolemia	0 (0%)	0 (0%)	0 (0%)	-
Hypertension plus hypercholesterolemia	3 (5%)	2 (6%)	1 (5%)	0.93
Diabetic mellitus plus hypercholesterolemia plus hypertension	5 (9%)	3 (8%)	2 (10%)	0.84
Physical activity				
Never	41 (73%)	25 (69%)	16 (80%)	0.65
Occasional	10 (18%)	7 (19%)	3 (15%)	
Regular	5 (9%)	4 (12%)	1 (5%)	
Dietary				
Omnivorous	44	29	15	0.86
Vegetarians	10	6	4	
Vegans	2	1	1	

SGA, Subjective Global Assessment; NRS, Nutritional Risk Score; Group A, well-nourished, group B, moderately undernourished, group C, severely undernourished; T3,4, tumor site and size.

**Table 3:** qRT-PCR primers.

mRNA	qRT-PCR Amplification Primers (5′-3′)
*FNDC5*	Forward: GGCTGCACTACAAACCCAAA
	Reverse: TTGTCATCTCCCAGGGCTTT
*GHRL*	Forward: AGTTCCCAAGGCATCCATCA
	Reverse: GGCAGGGATGTTAGCAAAGG
*TNT*	Forward: CGAAATGCATCAGTCAGCGA
	Reverse: TCCTTGCAAGCTTGTGTCAC
*Β-actin*	Forward: TCACCCACACTGTGCCCATCTACGA
	Reverse: CAGCGGAACCGCTCATTGCCAATGG

FNDC5, irisin; GHRL, ghrelin; TNT, titin.

**Table 4:** Characteristics of primers designed for the MSP.

mRNA		qRT-PCR Amplification Primers (5′-3′)	GC%	‘C’s
FNDC5 (irisin)	M	Forward: 5′-ATTTTTTAGTAGAAGAAGGATGTGC-3′ Reverse: 5′-AAATCTTAAAAAACACAAACTCGCT-3′	52.00 60.00	5 8
	U	Forward: 5′-TTTTAGTAGAAGAAGGATGTGTGG-3′ Reverse: 5′-AAATCTTAAAAAACACAAACTCACT-3′	58.33 60.00	4 8
Ghrelin	M	Forward: 5′-AGGGTTAAGGAGGAGTGTTTGTC-3′ Reverse: 5′-AAATAATTATTTCCCATATTCACGTC-3′	65.22 42.31	4 4
	U	Forward: 5′-AGGGTTAAGGAGGAGTGTTTGTT-3′ Reverse: 5′-AAATAATTATTTCCCATATTCACATC-3′	65.22 42.31	4 4
Titin	M	Forward: 5′-GAAAAGTTTATTTTTTTCGAATTGC-3′ Reverse: 5′-CTTTAACTTCCAAATCTTCAAAACG-3′	40.00 52.00	4 5
	U	Forward: 5′-GGAAAAGTTTATTTTTTTTGAATTGT-3′ Reverse: 5′-TTAACTTCCAAATCTTCAAAACACT-3′	40.74 52.00	4 5

M, primers designed for methylated sequences; U, primers designed for non-methylated sequences; ‘C’s, number of converted cytosines.

**Table 5:** Nutritional status of patients with HNSCC before surgery, the day of the start, and after 6 months of chemotherapy.

Nutritional Scale	T0	T3	T5	*p*-Value (Chi-Square Test)
SGA				
A	8 (14%)	14 (25%)	15 (27%)	0.52
B	37 (66%)	32 (57%)	33 (59%)	
C	11 (20%)	10 (18%)	8 (14%)	
NRS				
1	5 (9%)	6 (11%)	10 (18%)	0.91
2	3 (5%)	10 (18%)	11 (20%)	
3	34 (61%)	33 (59%)	28 (50%)	
>3	14 (25%)	7 (12%)	7 (12%)	

Data are presented as mean ± standard deviation; T0, the moment prior to the surgical procedure; T3, the day of the start of chemoradiotherapy; T5, six months after the completion of chemoradiotherapy; SGA, Subjective Global Assessment; NRS, Nutritional Risk Score; Group A, well-nourished, group B, moderately undernourished, group C, severely undernourished.

**Table 6:** Evaluation of the changes in the expression patterns of irisin, ghrelin, and titin using q-RT-PCR.

Type of Tissue	Time	Group According to the Nutrition Status	Log_2_ Fold Change		
			Irisin	Human Acylated Ghrelin	Titin
Tissue	-	General	(−)3.45 ± 0.87 ^1,2,3^	(−)2.34 ± 0.98 ^1,2,3^	(−)4.56 ± 0.45 ^1,2,3^
		A	(−)1.76± 0.98	(−)1.87 ± 0.93	(−)2.34 ± 0.16
		B	(−)3.21 ± 0.86	(−)2.13 ± 0.96	(−)3.98 ± 0.24
		C	(−)5.38 ± 0.77	(−)3.02 ± 1.05	(−)7.36 ± 0.21
Blood samples	T0	General	(−)4.56 ± 0.54 ^1,2,3^	(−)3.45 ± 0.23 ^2^	(−)3.46 ± 0.84 ^1,2,3^
		A	(−)2.87 ± 0.45	(−)3.12 ± 0.13	(−)1.98 ± 0.79
		B	(−)4.01 ± 0.65	(−)3.35 ± 0.24	(−)3.67 ± 0.98
		C	(−)6.80 ± 0.51	(−)3.98 ± 0.31	(−)4.73 ± 0.76
	T1	General	(−)4.11 ± 0.92^1,2,3^	(−)3.41 ± 0.12 ^1,2,3^	(−)3.54 ± 0.98 ^1,2,3^
		A	(−)2.98 ± 0.87	(−)1.59 ± 0.11	(−)2.00 ± 0.99
		B	(−)4.12 ± 0.99	(−)2.98 ± 0.13	(−)3.87 ± 0.91
		C	(−)5.23 ± 0.91	(−)5.66 ± 0.12	(−)4.75 ± 1.04
	T2	General	(−)3.14 ± 0.23 ^1,2,3^	(−)3.21 ± 0.18 ^1,2,3^	(−)3.45 ± 0.19 ^1,2,3^
		A	(−)2.78 ± 0.24	(−)1.84 ± 0.19	(−)2.01 ± 0.21
		B	(−)2.97 ± 0.21	(−)3.44 ± 0.19	(−)3.51 ± 0.20
		C	(−)3.76 ± 0.24	(−)4.35 ± 0.16	(−)4.83 ± 0.16
	T3	General	(−)2.34 ± 0.19 ^1,2,3^	(−)2.45 ± 0.12 ^1,2,3^	(−)2.12 ± 0.18 ^1,2,3^
		A	(−)1.06 ± 0.16	(−)1.87 ± 0.12	(−)1.56 ± 0.25
		B	(−)2.18 ± 0.21	(−)2.50 ± 0.13	(−)1.98 ± 0.16
		C	(−)3.78 ± 0.20	(−)2.98 ± 0.11	(−)2.82 ± 0.13
	T4	General	(−)2.11 ± 0.65 ^1,2,3^	(−)2.99 ± 0.87 ^1,2,3^	(−)2.45 ± 0.77 ^1,2,3^
		A	(−)1.43 ± 0.45	(−)1.21 ± 0.98	(−)1.77 ± 0.80
		B	(−)2.12 ± 0.87	(−)3.80 ± 0.71	(−)2.13 ± 0.67
		C	(−)2.78 ± 0.56	(−)3.96 ± 0.92	(−)3.45 ± 0.84
	T5	General	(−)1.56 ± 0.29 ^1,2,3^	(−)1.21 ± 0.98	(−)2.13 ± 0.99 ^1^
		A	(−)1.12 ± 0.23	(−)1.11 ± 1.01	(−)2.01 ± 1.04
		B	(−)1.58 ± 0.31	(−)1.23 ± 0.89	(−)2.07 ± 1.01
		C	(−)1.87 ± 0.33	(−)1.19 ± 1.04	(−)2.31 ± 0.92

Data are presented as mean ± standard deviation; T0, the moment prior to the surgical procedure; T1, the first day after the surgical procedure; T2, the seventh day after the surgical procedure; T3, the day of the start of chemoradiotherapy; T4, moment of completion of chemoradiotherapy; T5, six months after the completion of chemoradiotherapy; A, well-nourished group, B, moderately undernourished group, C, severely undernourished group; ^1^, *p* < 0.05 (A vs. B); ^2^, *p* < 0.05 (A vs. C); ^3^, *p*< 0.05 (B vs. C).

**Table 7:** The degree of methylation in the studied and control groups.

Type of Tissue	Time	Irisin		Human Acylated Ghrelin		Titin	
		Methylated	Non-Methylated	Methylated	Non-Methylated	Methylated	Non-Methylated
Cancer tissue	-	55 (98%)	1 (2%)	55 (98%)	1 (2%)	54 (96%)	2 (4%)
Margin tissue	-	1 (2%)	55 (98%)	1 (2%)	55 (98%)	2 (4%)	54 (96%)
Blood samples	C	1 (2%)	69 (99%)	0 (0%)	70 (100%)	2 (4%)	68 (96%)
	T0	56 (100%)	0 (0%)	56 (100%)	0 (0%)	56 (100%)	0 (0%)
	T1	56 (100%)	0 (0%)	56 (100%)	0 (0%)	56 (100%)	0 (0%)
	T2	56 (100%)	0 (0%)	56 (100%)	0 (0%)	56 (100%)	0 (0%)
	T3	56 (100%)	0 (0%)	56 (100%)	0 (0%)	56 (100%)	0 (0%)
	T4	56 (100%)	0 (0%)	56 (100%)	0 (0%)	56 (100%)	0 (0%)
	T5	56 (100%)	0 (0%)	56 (100%)	0 (0%)	56 (100%)	0 (0%)

C, control; T0, moment prior to the surgical procedure; T1, first day after the surgical procedure; T2, seventh day after the surgical procedure; T3, day of start of chemoradiotherapy; T4, moment of completion of chemoradiotherapy; T5, six months after the completion of chemoradiotherapy.

**Table 8:** Concentration of irisin, ghrelin, and titin in tissue and blood obtained using ELISA.

Type of Tissue	Time	Group	Irisin [ng/mL]	Human Acylated Ghrelin [pg/mL]	Titin [pg/mL]
Margin tissue (control)	-	-	103.24 ± 0.76	141.12 ± 2.23	51.11 ± 1.23
Cancer tissue	-	General	21.42 ± 0.19 ^1,2,3^	37.12 ± 1.23 ^1,2,3^	24.56 ± 0.12 ^1,2,3^
		A	24.56 ± 0.23	43.21 ± 1.56	27.98 ± 0.13
		B	21.09 ± 0.21	39.24 ± 1.21	27.69 ± 0.11
		C	15.9 ± 0.13	28.91 ± 0.92	18.01 ± 0.12
Blood samples	-	Control	78.17 ± 0.98	189.09 ± 1.34	102.98 ± 1.45
	T0	General	21.14 ± 0.44 ^1,2,3^	21.98 ± 0.13 ^1,2,3^	28.98 ± 0.12 ^1,2,3^
		A	25.19 ± 0.46	27.87 ± 0.12	34.87 ± 0.12
		B	21.12 ± 0.42	20.55 ± 0.15	33.09 ± 0.15
		C	17.10 ± 0.44	17.52 ± 0.12	18.98 ± 0.09
	T1	General	18.76 ± 0.14 ^1,2,3^	22.13 ± 0.87 ^1,2,3^	28.09 ± 0.92 ^1,2,3^
		A	21.09 ± 0.16	25.14 ± 0.98	32.98 ± 0.98
		B	19.30 ± 0.12	23.19 ± 0.54	26.87 ± 0.95
		C	15.90 ± 0.14	18.06 ± 1.09	24.42 ± 0.83
	T2	General	19.09 ± 0.76 ^1,2,3^	22.23 ± 0.99 ^1,2,3^	30.01 ± 0.82 ^1,2,3^
		A	21.76 ± 0.85	25.09 ± 1.02	35.98 ± 0.54
		B	19.87 ± 0.54	22.19 ± 0.97	27.13 ± 0.86
		C	15.64 ± 0.89	19.41 ± 0.95	26.92 ± 1.06
	T3	General	24.89 ± 0.12 ^1,2,3^	29.98 ± 0.45 ^1,2,3^	34.98 ± 1.33 ^1,2,3^
		A	29.17 ± 0.15	36.78 ± 0.23	41.11 ± 1.54
		B	23.45 ± 0.09	31.09 ± 0.65	34.56 ± 1.01
		C	22.05 ± 0/12	22.07 ± 0.47	29.27 ± 1.44
	T4	General	25.09 ± 0.13 ^1,2,3^	29.01 ± 0.89 ^1,2,3^	35.61 ± 0.99 ^1,2,3^
		A	27.18 ± 0.12	35.11 ± 0.76	41.23 ± 1.21
		B	23.56 ± 0.14	30.98 ± 0.99	36.78 ± 0.43
		C	24.53 ± 0.13	20.94 ± 0.92	28.82 ± 1.33
	T5	General	25.15 ± 0.37 ^1,2,3^	29.54 ± 1.65 ^1,2,3^	35.18 ± 1.41 ^1,2,3^
		A	27.11 ± 0.30	34.56 ± 1.76	38.98 ± 1.43
		B	22.98 ± 0.41	29.18 ± 1.00	32.11 ± 1.41
		C	25.36 ± 0.40	24.88 ± 1.11	34.45 ± 1.39

Data are presented as mean ± standard deviation; T0, the moment prior to the surgical procedure; T1, the first day after the surgical procedure; T2, the seventh day after the surgical procedure; T3, the day of the start of chemoradiotherapy; T4, moment of completion of chemoradiotherapy; T5, six months after the completion of chemoradiotherapy; A, well-nourished group, B, moderately undernourished group, C, severely undernourished group; ^1^, *p* < 0.05 (A vs. B); ^2^, *p* < 0.05 (A vs. C); ^3^, *p* < 0.05 (B vs. C).

**Table 9:** Blood group distribution between study and control groups.

Blood Group	A n (%)	B	AB	0
Study group	34 (61)	6 (11)	3 (5)	13 (23)
Control group	27 (38)	6 (9)	3 (4)	34 (49)
Odds ratio	2.46	1.28	1.26	0.32

## Data Availability

All data generated or analyzed during this study are included in this published article.
